# Sequencing of ATCC 19951-B2 reveals *Enterococcus* bacteriophage misidentified as *Streptococcus* sp. phage

**DOI:** 10.1128/mra.01280-25

**Published:** 2026-06-22

**Authors:** Nicolas Fournier, Steven S. Theriault, Tasia Joy Lightly

**Affiliations:** 1 Cytophage Technologies Limited, Winnipeg, Manitoba, Canada; 2 Department of Microbiology, University of Manitoba 8664, Winnipeg, Manitoba, Canada; University of Wisconsin-Madison, Madison, Wisconsin, USA

**Keywords:** bacteriophages, bacteriophage genetics

## Abstract

With the purpose of finding phages that can target Manitoba farm isolates of *Streptococcus suis*, we began the characterization of a control strain, *Streptococcus* sp. bacteriophage 118 (ATCC 19951-B2). Here we describe the genome sequencing and reclassification of the 154,261-bp virus as an *Enterococcus* phage.

## ANNOUNCEMENT

*Streptococcus suis* is a bacterial pathogen on Manitoba farms that causes disease in humans and swine (1). In the process of hunting for *S. suis* phages for the development of farm therapies, the *Streptococcus* strain AE-126 (ATCC 19951) and *Streptococcus* sp. bacteriophage 118 (ATCC 19951-B2), hereafter referred to as AE-126 and Phage 118, were obtained from the American Type Culture Collection (ATCC) as isolation controls. Lack of susceptibility to alternative hosts led to sequencing of both stocks, revealing that AE-126 is an *Enterococcus faecium* strain, not *Streptococcus* sp., and that Phage 118 is an *Enterococcus* phage. Previously performed nucleic acid hybridization studies led to the reclassification of *Streptococcus faecium* to *Enterococcus faecium* (2)*,* which may inform the current catalog mis-designation.

To propagate ATCC 19951-B2, 300 µL of an overnight culture of AE-126 was grown in brain heart infusion (BHI) medium, and 100 µL of the phage suspension was inoculated into 3 mL of BHI with 0.4% agarose overlay and plated on BHI with 0.8% agarose underlay. Phage suspensions were maintained in SMG buffer (50 mM Tris, 100 mM NaCl, 17 mM MgSO_4_, 0.01% gelatin, pH 7.5). Plates were incubated at 37°C with 5% CO_2_.

**Fig 1:**
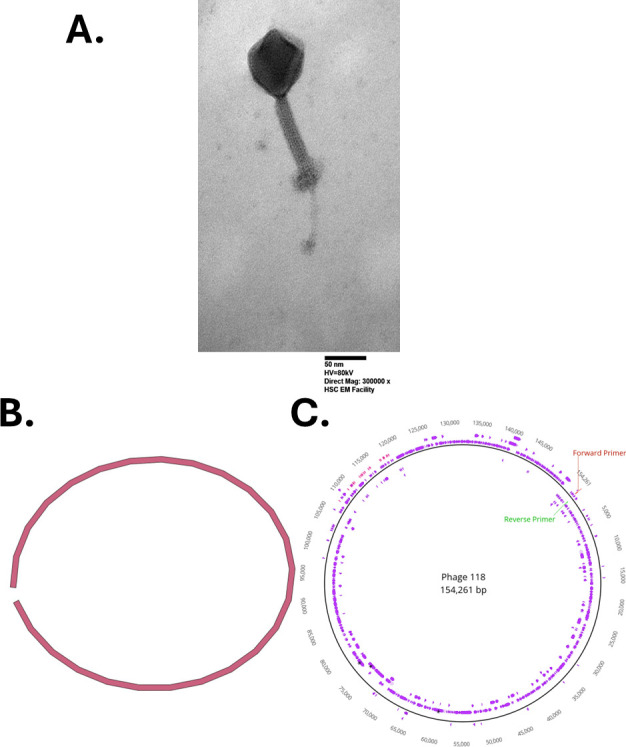
Morphology and genomic architecture of Phage 118. (**A**) Transmission electron micrograph image displaying the capsid, tail, and tail spike of phage 118. The sample was inactivated using ammonium acetate and stained using 1% (wt/vol) uranyl acetate. Imaged using JEOL JEM 1200 EXTEMSCAN transmission electron microscope at 80 kV by CCEM. Image is displayed at a 300,000× direct magnification with a 50 nm scale bar. (**B**) Bandage plot representing the *de novo* assembly of the phage genome, indicating one circular contig with successful closure and continuity. (**C**) Annotated genome map of Phage 118 (154,261 bp), highlighting open reading frames (ORFs) in purple and predicted tRNA genes in pink. Forward and reverse primers (red and green, respectively) used to confirm genome circularity are also indicated.

Phage DNA was purified from a 100 kDa Amicon centrifugal filter concentrated sample (Millipore Sigma, Canada) using a phenol-chloroform extraction (3). The DNA sample was included in a sequencing multiplex library prepared using the native barcoding genomic DNA (with EXP-NBD104, EXP-NBD114, and SQK-LSK109) protocol provided by Oxford Nanopore Technologies. The final library size was selected for using the short fragment buffer (SFB) wash solution. Sequencing was performed using the MinION platform with the R9 FLO-MIN106. Sequences were base-called and demultiplexed using the Guppy software v4.0.9 (Oxford Nanopore Technologies). Using default parameters, the genome was assembled into a single contig from 16,000 reads with an N_50_ of 12,239 and a minimum overlap of 1,000 using the Flye v2.9.6-b1802 assembly tools on the Galaxy v2.9.6 platform (4). The genome assembly, along with the Galaxy *Bandage Image* v2022.09+galaxy4 software, predicted a single, gapless circular genome with an assembly coverage of 154× (Fig. 1B). Using Minimap2 v2.24-r1122, 83.2% of reads were aligned to the assembled FASTA file. Amplified PCR product of the raw contig ends further suggested genome circularization (forward primer 5′-TCAATTTATTTTAAGCTATTTTAAAGC-3′ and reverse primer 5′-AAATAAATAAGAAACTACACGGG-3′). PCR in a 20 µL volume used 10 µL of GoTaq Green Master Mix (Promega), 0.2 µM of primers, and 1 µL of boiled phage lysate stock. Reactions were run in a Biometra Tone thermocycler using the following parameters: 95°C for 5 min; 95°C for 30 s; 58°C for 30 s; 72°C for 30 s: repeat 30 cycles; 72°C 5 min. Amplicon size (361 bp) was confirmed through gel electrophoresis. Annotations of contig were performed through the Bacterial and Viral Bioinformation Resource Center (BV-BRC v 3.49.1) using the *Genome Annotation* tool with the *Bacteriophage* Annotation Recipe (5). BLASTn searches for closest nucleotide relatives were done with the assembled FASTA file using default parameters to determine taxonomy (6). Phage 118 morphology (Fig. 1A) was determined through negatively stained samples with 1% (wt/vol) uranyl acetate and imaged using a JEOL JEM 1200 EXTEMSCAN transmission electron microscope at the Canadian Centre for Electron Microscopy (CCEM).

Phage 118 is a 154,261-bp *Herellephage* with 37.1% G+C content. Annotation predicted 24 tRNA genes and 387 protein-coding genes (Fig. 1C). Closely related nucleotide sequences are shown in Table 1.

**TABLE 1:** Pairwise similarity of BLASTn results to Phage 118

Name	Accession number	% Similarity	Size (bp)
Phage 118	PX399211	N/A^*a*^	154,261
*Enterococcus* phage 113	MZ147816.1	99.69	155,715
*Enterococcus* phage Porthos	LR990835.2	98.57	151,703
*Enterococcus* phage phiSHEF16	OL799260.1	98.32	151,935
*Enterococcus* phage iF6	MT909815.1	97.96	156,592

^*a*^ N/A is not applicable as the percent similarity is compared to that of Phage 118.

## Data Availability

The data of Phage 118 genome sequence and reads are available at GenBank accession no. PX399211 and Sequence Read Archive (SRA) no. PRJNA1307615.
